# Genome-Wide DNA Methylation Profile in Whole Blood of Patients With Chronic Spontaneous Urticaria

**DOI:** 10.3389/fimmu.2021.681714

**Published:** 2021-09-03

**Authors:** Yumeng Qi, Liming Zhang, Xiaonan Yang, Biao Tang, Ting Xiao

**Affiliations:** ^1^Department of Dermatology, The First Hospital of China Medical University, National Health Commission Key Laboratory of Immunodermatology, Key Laboratory of Immunodermatology of Ministry of Education, Shenyang, China; ^2^Sinotech Genomics Co., Ltd, Shanghai, China

**Keywords:** chronic spontaneous urticaria, DNA methylation, epigenetics, autoimmune, whole blood

## Abstract

**Background:**

Chronic spontaneous urticaria (CSU) is a common autoimmune skin disease. Little is known about the role of epigenetics in the pathogenesis of CSU. This study aimed to investigate genome-wide DNA methylation profile in whole blood of patients with CSU.

**Patients and Methods:**

Genome-wide DNA methylation levels in whole blood samples of 95 Chinese Han ethnicity adult CSU patients and 95 ethnicity-, age- and sex-matched healthy controls were analyzed using Illumina 850K methylation chip. The differentially methylated genes (DMGs) were screened out and then functionally annotated by the gene ontology and the Kyoto encyclopedia of genes and genomes databases.

**Results:**

A total of 439 differentially methylated positions (DMPs) (*p* < 0.01 and |Δ*β*| ≥ 0.06) were identified with 380 hypomethylated and 59 hypermethylated. The average global DNA methylation levels of the 439 DMPs in the CSU patients were significantly lower than those in the healthy controls (*p* < 0.001). The distribution of the 439 DMPs was wide on chromosome 1 to 22 and chromosome X. Chromosome 6 embodied the largest number of DMPs (*n* = 51) and their annotated genes were predominantly related to autoimmunity. The 304 annotated DMGs were mainly enriched in autoimmune disease- and immune-related pathways. A total of 41 DMPs annotated to 28 DMGs were identified when *p* < 0.01 and |Δ*β*| ≥ 0.1. Of the 28 DMGs, HLA-DPB2, HLA-DRB1, PPP2R5C, and LTF were associated with autoimmunity. CSU cases with elevated total IgE, positive anti-thyroid peroxidase IgG autoantibodies, positive anti-thyroglobulin IgG autoantibodies, angioedema, UASday > 4, or recurrent CSU showed phenotype-specific DMPs as compared with cases with normal total IgE, negative anti-thyroid peroxidase IgG autoantibodies, negative anti-thyroglobulin IgG autoantibodies, no angioedema, UASday ≤ 4, or non-recurrent CSU respectively.

**Conclusion:**

This study shows a distinct genome-wide DNA methylation profile in Chinese Han ethnicity adult CSU patients and indicates a role of epigenetics in the pathogenesis of CSU. The predominant enrichment of the CSU-associated DMGs in immunological pathways provides supportive evidence for the immunopathogenesis of CSU. Future research on the CSU-associated DMPs and DMGs will help discover potential therapeutic targets for CSU.

## Introduction

Chronic spontaneous urticaria (CSU) is a common mast cell-driven allergic dermatosis characterized by spontaneous wheals, angioedema, or both lasting for at least 6 weeks (1). CSU is a polygenic autoimmune skin disease and has a multi-factorial pathogenesis. Although previous candidate gene studies have reported some CSU-associated genetic variants (**Supplementary Table 1**), so far, no widely accepted susceptibility or risk loci of CSU have been identified. The interaction of genetic and environmental factors in the pathogenesis of CSU suggests a possibility of epigenetic dysregulation as a contributor of development of CSU. Although autoimmune theories of skin mast cell activation in CSU have been widely accepted (2), epigenetic pathomechanisms in CSU remain unclear.

CSU has been reported to be associated with autoimmune diseases including autoimmune thyroid diseases (ATDs), inflammatory bowel diseases (IBDs), rheumatoid arthritis (RA), type I diabetes (TID), and vitiligo (3). Dysregulated DNA methylation is related to the pathogenesis of systemic lupus erythematosus (SLE) and RA (4) and differentially methylated genes (DMGs) have been used as biomarkers for disease activity, treatment response and prognosis of SLE, ATDs and IBDs (5–7).

To the best of our knowledge, so far, no genome-wide DNA methylation study of CSU has been published. In this study, we performed a genome-wide DNA methylation analysis using whole blood samples of a Chinese Han ethnicity adult CSU population. The potential target signal pathways of the DMGs were bioinformatically analyzed.

## Materials And Methods

### Study Population

Ninety-five Chinese Han ethnicity adult outpatients with active CSU diagnosed according to the criteria of the EAACI/GA^2^LEN/EDF/WAO guideline were enrolled from June 2014 to April 2018 (8). Recurrent CSU was defined as occurrence of CSU after absence of symptoms for at least 1 year without administering any medications. Patients with chronic inducible urticaria, urticarial vasculitis, or CSU patients who had been treated by systemic corticosteroids and/or immunosuppressants were excluded. CSU patients who simultaneously suffered from active autoimmune diseases or allergic diseases were excluded. Demographic data, medical histories, and clinical data were obtained using a questionnaire on presentation. Physical examinations and urticaria activity score of 1 day (UASday) were performed and evaluated by a dermatologist and a postgraduate. Ninety-five ethnicity-, age-, and sex-matched healthy controls with serum total IgE (tIgE) <100 IU/ml, negative specific IgEs (sIgEs), negative anti-thyroid peroxidase (TPO) IgG autoantibodies (AAbs), and negative anti-thyroglobulin (TG) IgG AAbs were recruited. This study was approved by the institutional ethics committee of The First Hospital of China Medical University. Each participant signed a written informed consent.

### Therapeutic Regimen

We used a therapeutic regimen as previously described (9). Second-generation H_1_-antihistamines (sgAHs) in licensed doses were used as the first-step treatment. An increase in the dose of the sgAHs to a maximum of fourfold dose or combinations of sgAHs up to a fourfold equivalent dose comprised the second-step treatments. H_2_-antihistamines (ranitidine or famotidine) and/or leukotriene receptor antagonist (montelukast) was added as the third-step treatments. Before complete control was achieved, or during the dose-increment stage, patients were assessed weekly and UASday was recorded at each visit. CSU cases failing to be completely controlled by fourfold doses or fourfold equivalent doses of sgAHs for at least 2 weeks were designated as sgAHs-refractory cases.

### Detection of Serum tIgE, sIgEs, and Anti-Thyroid IgG AAbs

Serum levels of tIgE (Euroimmun, Lübeck, Germany) were measured using ELISA kits according to the manufacturer’s protocols (normal range: 0–100 IU/ml). Serum sIgEs were detected using immunoblotting (Euroimmun) as previously described (10). The detected sIgEs included 8 inhalant sIgEs and 10 food sIgEs. A test result of < 0.70 kU/L was considered negative. Serum levels of anti-TPO (normal range: 0.00–5.61 IU/ml) and anti-TG (normal range: 0.00–4.11 IU/ml) IgG AAbs were measured according to the manufacturer’s protocols (Abbott Park, Middletown, USA).

### Genome-Wide DNA Methylation Measurement

Genomic DNA was isolated from whole blood using QIAamp DNA MiniKit (Qiagen, Hilden, Germany). The purity and concentration of DNA was estimated using Nanodrop 2000 (Thermo)/Quibt 3.0. Then, 500 ng DNA of each sample was used to bisulfite converted using EZ DNA Methylation Kits (Zymo Research, USA), and the converted products were put into Illumina Infinium Human Methylation 850K BeadChip (Illumina Inc, CA, USA) in accordance with the manufacturer’s guidelines and protocol.

### Genome-Wide DNA Methylation Array Analysis

The array data of Illumina methylation chip (.IDAT files) were analyzed using ChAMP package in the R software for deriving the methylation level. Firstly, CpG probe filtering of raw data was performed by removing (1) CpG probes with a detection *p* ≥ 0.01 in 1% of samples; (2) CpG probes with bead count < 3 in 5% of samples; (3) non-CpG probes; (4) CpG probes with single-nucleotide polymorphisms (SNPs) relation; and (5) CpG probes that aligned to multiple locations. Secondly, beta-mixture quantile normalization adjustments for correcting type I and type II probe design bias were used to standardize the methylation data (11). Thirdly, we used singular value decomposition (SVD) analysis to analyze the batch effect caused by BeadChip slide and array, then applied Combat to correct this batch effect (12). SVD analysis was also used to analyze the effect of confounders including age, sex and smoking status on the DNA methylation of the samples.

As genomic DNA was extracted from clustering leukocytes, we estimated leukocyte compositions including CD4^+^ T lymphocytes, CD8^+^ T lymphocytes, natural killer (NK) cells, B lymphocytes, monocytes, and granulocytes, by the RefbaseEWAS method for inferring changes in the distribution of leukocytes between the CSU patients and the healthy controls using DNA methylation signatures, in combination with a previously obtained external validation set consisting of signatures from purified leukocyte samples (13).

Differential methylated CpG positions were calculated by differentially methylated position (DMP) analysis of ChAMP, and the corrected *p* values were computed using the Benjamini–Hochberg method (14). The methylation level of each CpG probe was denoted as a *β* value. The Δ*β* of each CpG site represents the difference in average *β* values of the CSU patients and the healthy controls. A CpG site with |Δ*β*| ≥ 0.06 and *p* < 0.01 was considered as a DMP or a differentially methylated CpG site. A CpG site was considered hypermethylated if Δ*β* ≥ 0.06 or hypomethylated when Δ*β* ≤ −0.06.

Since there are many different cell types in the whole blood, the difference caused by the change of a single cell type is not significant in the whole blood. Therefore, the cutoff value for *β* of the whole blood sample is generally low. In previous literature, 0.1 was commonly used as a cutoff value for *β* in peripheral blood samples. In this study, we used 0.06 as the cutoff value for *β* (simultaneously *p* < 0.01) for bioinformatic analysis and 0.1 as the cutoff value for *β* for single CpG site analysis. Moreover, 0.06 was used as the cutoff value for *β* to avoid the chip-based error of 0.05 in this study.

### Bioinformatic Analysis

To explore the potential biological functions of target genes of DMPs or differentially methylated CpG sites, we performed gene ontology enrichment analysis including molecular function, cellular component, and biological process using the Annotation, Visualization, and Integrated Discovery (DAVID) database as well as pathway enrichment analysis using the Kyoto Encyclopedia of Genes and Genomes (KEGG) database. A *p* value < 0.05 was used as the threshold to select the significantly enriched gene ontology terms and pathways.

### Statistical Analysis

Data were processed using GraphPad Prism 8.0 and SPSS 23.0. Normally distributed continuous variables were compared by using Student’s *t*-test while Mann–Whitney *U*-test was used for comparison of non-normally distributed continuous variables. The associations between categorical variables were analyzed using Pearson’s chi-squared test. *p* < 0.05 was considered statistically significant. We use *p*-value in the test of the entire article rather than corrected *p*-value.

## Results

### Description of the Study Population

Demographic, clinical, and laboratory data of the 95 CSU patients and the 95 healthy controls are summarized in **Supplementary Table 2**. The 95 CSU patients achieved complete control using different treatment regimens. Among the 95 patients, 36 were refractory. The therapeutic regimens and characteristics of the 36 refractory cases and 59 non-refractory cases are listed in **Supplementary Tables 3–5**.

### Genome-Wide DNA Methylation Profile of the CSU Patients

**Figure 1:**
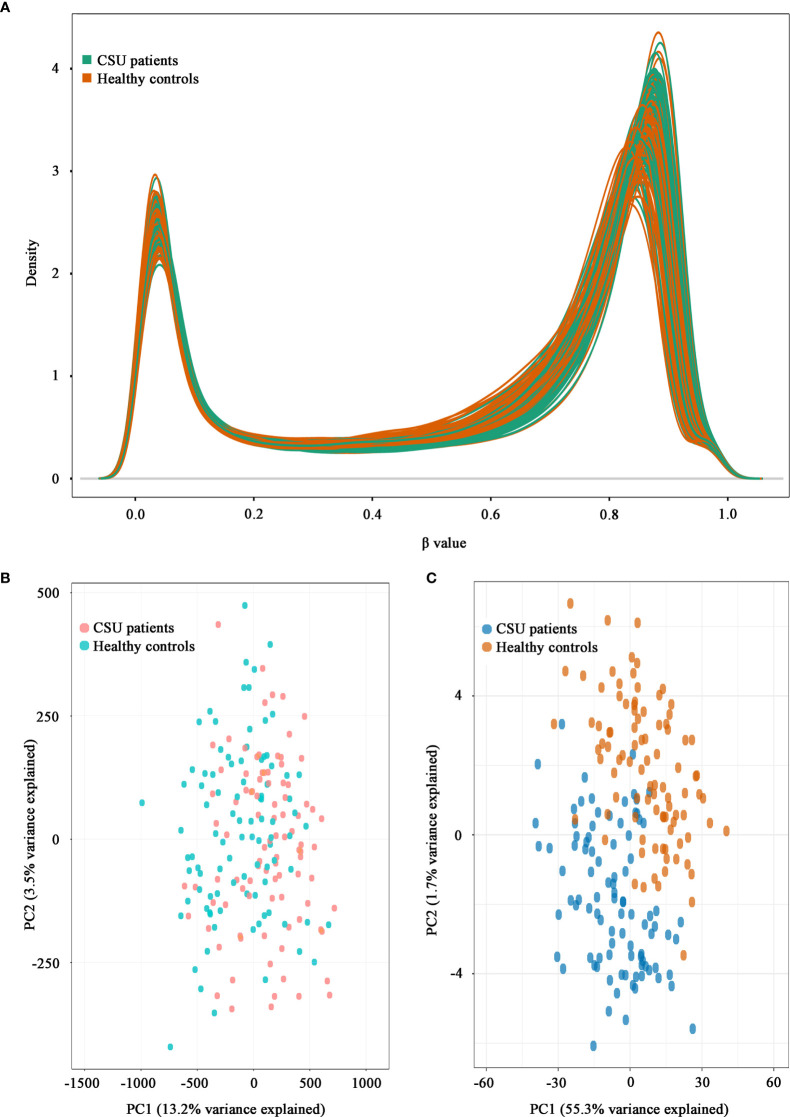
Density plot and principal component analysis (PCA) plot. **(A)** Density plot. The methylation levels of the 95 CSU patients (green lines) and the 95 healthy controls (red lines) are expressed as *β* values. **(B)** PCA plot based on all 832,347 CpG sites. No discernable separation is observed between the CSU patients (red dots) and the healthy controls (green dots). PC1, principal component 1 (13.2% variance explained); PC2, principal component 2 (3.5% variance explained). **(C)** PCA plot based on 439 differentially methylated positions (DMPs). Two different clusters are observed between the CSU patients (blue dots) and the healthy controls (red dots). PC1, principal component 1 (55.3% variance explained); PC2, principal component 2 (1.7% variance explained).

A total of 832,347 CpG sites were analyzed following quality filtering and data normalization. The raw data will be available at https://www.biosino.org/node with accession number OEP002482 upon approval by Ministry of Science and Technology, China. Density plot showed relatively similar methylation levels in the samples from the CSU patients and the healthy controls (**Figure 1A**). Principal component analysis (PCA) based on all 832,347 CpG sites did not reveal any discernable separation of the CSU patients from the healthy controls (**Figure 1B**). Leukocyte composition analysis showed that the percentages of CD8^+^ T lymphocytes, NK cells, and monocytes were significantly lower in the CSU patients than those in the healthy controls, whereas the percentage of granulocytes was significantly higher in the CSU patients than that in the healthy controls (**Table 1**).

**Table 1:** Comparisons of the percentages of the leukocyte components in the CSU patients and the healthy controls.

	CSU patients (*n* = 95)		Healthy controls (*n* = 95)		*p*-value^#^
	Mean (%)	SD	Mean (%)	SD	
CD8^+^ T cells	9.69	3.51	12.09	4.13	<0.0001
CD4^+^ T cells	12.99	3.84	13.52	4.38	0.38
NK cells	3.92	2.70	5.48	2.97	<0.0001
B cells	4.92	2.06	4.64	1.62	0.30
Monocytes	5.88	1.91	6.51	1.91	0.02
Granulocytes	64.06	8.46	59.14	8.14	<0.0001

CSU, chronic spontaneous urticaria; SD, standard deviation; NK cells, natural killer cells.

^#^Student’s t-test.

**Figure 2:**
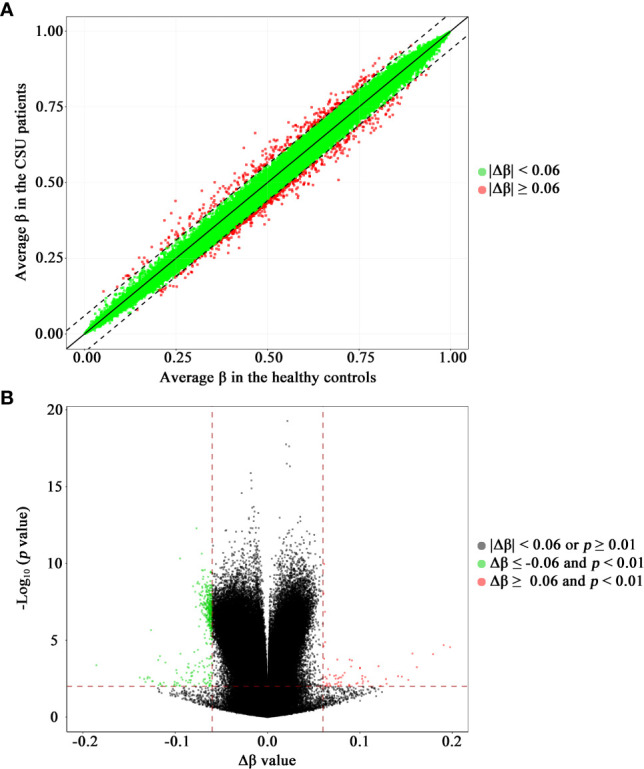
Scatter plot and volcano plot of the 832,347 CpG sites between the CSU patients and the healthy controls. **(A)** Scatter plot. This scatter plot indicates the overall correlation of the average methylation levels between the CSU patients and the healthy controls. The ordinate and abscissa represent the average *β* value for each CpG site of the 95 CSU patients and the 95 healthy controls, respectively. A CpG site with |Δ*β*| < 0.06 and a CpG site with |Δ*β*| ≥ 0.06 is signed by a green circle and a red circle, respectively. **(B)** Volcano plot. The ordinate represents the *p*-value (−log10 scale) for each CpG site and the abscissa represents the Δ*β* value of each CpG site. Thresholds are shown as dashed lines. The vertical red dashed lines represent 6% change in Δ*β* values. The horizontal red dashed line represents the significant cutoff of *p* = 0.01. A gray circle represents a CpG site with |Δ*β*| < 0.06 or *p* ≥ 0.01. A green circle represents a hypomethylated differentially methylated position (DMP) with Δ*β* ≤ −0.06 and *p* < 0.01. A red circle represents a hypermethylated DMP with Δ*β* ≥ 0.06 and *p* < 0.01. CSU, chronic spontaneous urticaria.

The methylation levels of the CpG sites in the CSU patients and the healthy controls were strongly correlated (*R* ^2^ = 0.999, *p* < 0.0001, **Figure 2A**). Compared with the 95 healthy controls, 439 DMPs (*p* < 0.01 and |Δ*β*| ≥ 0.06) were identified in the 95 CSU patients. Among the 439 DMPs, 380 (86.56%) were hypomethylated (*p* < 0.01 and Δ*β* ≤ −0.06) and 59 (13.44%) were hypermethylated (*p* < 0.01 and Δ*β* ≥ 0.06) (**Supplementary Table 6**). Volcano plot of the differential DNA methylation analysis showed obviously that the number of hypomethylated DMPs were far more than that of hypermethylated DMPs (**Figure 2B**). Although there was some overlap between the CSU patients and the healthy controls, the PCA based on the 439 DMPs showed two different clusters between the CSU patients and the healthy controls (**Figure 1C**).

**Figure 3:**
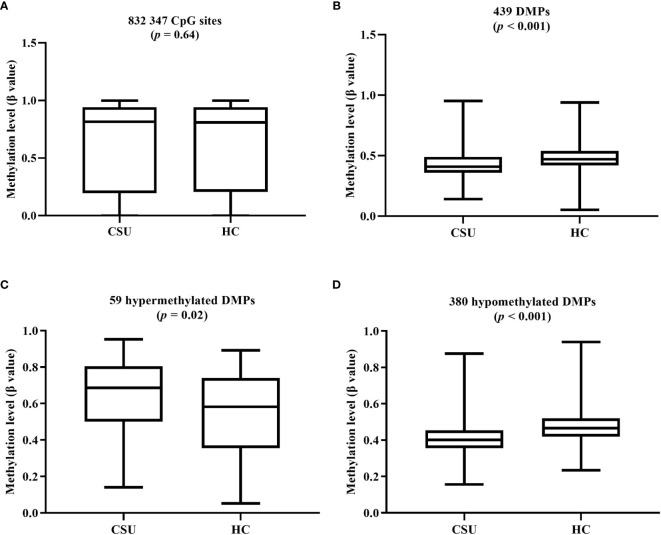
**(A)** The average global levels of DNA methylation for the 832,347 CpG sites between the CSU patients and the healthy controls (HCs) showed no significant difference (*p* = 0.64). **(B)** The average methylation levels (*β* values) of the 439 differentially methylated positions (DMPs) in the 95 CSU patients were significantly lower than those in the 95 HCs (*p* < 0.0001). **(C)** With regard to the 59 hypermethylated DMPs, the average methylation levels of the 95 CSU patients were significantly higher than those of the HCs (*p* = 0.02). **(D)** Concerning the 380 hypomethylated DMPs, the average methylation levels of the 95 CSU patients were significantly lower than those of the HCs (*p* < 0.0001).

The average global DNA methylation levels of the 832,347 CpG sites between the 95 CSU patients and the 95 healthy controls showed no significant difference (**Figure 3A**). The average global DNA methylation levels of the 439 DMPs in the 95 CSU patients were significantly lower than those in the 95 healthy controls (*p* < 0.0001) (**Figure 3B**). The average methylation levels of the 59 hypermethylated DMPs in the 95 CSU patients were significantly higher than those in the 95 healthy controls (*p* = 0.02) and the average methylation levels of the 380 hypomethylated DMPs in the 95 CSU patients were significantly lower than those in the 95 healthy controls (*p* < 0.0001) (**Figures 3C, D**).

We used SVD analysis to analyze the effect of confounders including age, sex, and smoking status on the DNA methylation of the samples. From the statistical *p* values corresponding to the color blocks (**Supplementary Figure 1**), age, sex and smoking status (28 CSU patients and 4 healthy controls were active smokers) showed significant correlation with DNA methylation of the samples. Then, we searched in PubMed for previously reported age-, sex-, and smoking-related CpGs and used the online tool of Venny 2.1 assisted by manual checking to identify overlapped DMPs. No overlapped DMP was found between the 439 DMPs discovered in our study and previously reported age-related CpGs (15) or smoking-related CpGs (16). In the 439 DMPs, 5 CpGs (cg04732279, cg12873598, cg16158408, cg11887420, and cg18105467) were located on chromosome X. However, none of the 5 CpGs was overlapped with previously reported sex-related CpGs (17). Therefore, the effect of confounders including age, sex, and smoking status on the DNA methylation of the 439 DMPs discovered in our study was excluded.

**Figure 4:**
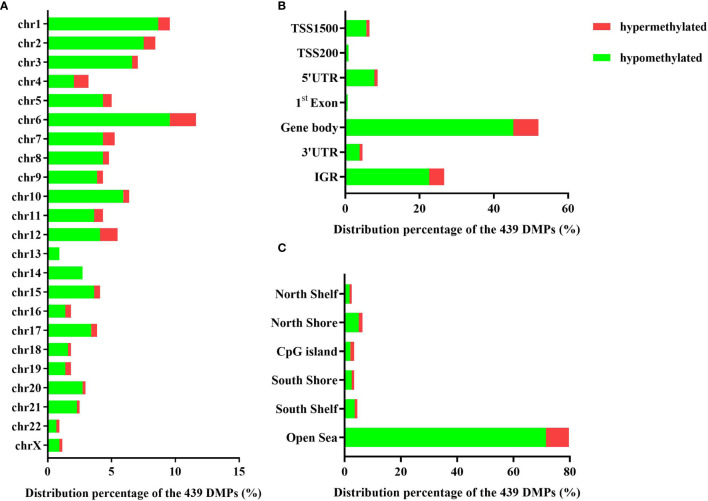
**(A)** The distribution percentages of the 439 differentially methylated positions (DMPs) on different chromosomes. **(B)** The distribution percentages of the 439 DMPs on different gene regions including TSS1500, TSS200, 5’UTR, 1st Exon, gene body, 3’UTR, and intergenic regions (IGR). The red bars represent hypermethylated DMPs whereas the blue bars represent hypomethylated DMPs. **(C)** The distribution percentages of the 439 DMPs in different CpG island regions including North Shelf, North Shore, CpG island, South Shore, South Shelf, and Open Sea.

The 439 DMPs in the 95 CSU patients were distributed widely on chromosome 1 to 22 and chromosome X (**Figure 4A**). Among the 23 chromosomes, chromosome 6 contained the largest number of DMPs (*n* = 51), among which 15 DMPs-annotated genes *TRIM38, MAP3K5, ARG1, C6orf106, SRPK1, MAPK14, COL11A2, HLA-DRB1, HLA-DPB2, HLA-DQB1, HLA-C, C6orf10, BAT3, HLA-DPB1*, and *COL21A1* were definitely involved in autoimmunity in the literature. Moreover, the comparisons between the distribution intensity of the 439 DMPs on each chromosome and the corresponding distribution intensity of the 832,347 CpG sites on each chromosome showed that only the ratio of chromosome 6 had a significant elevation (**Table 2**). With regard to corresponding gene regions (**Figure 4B**), 79 (16.70%) DMPs were located in proximal promoter regions including TSS1550 (200–1,500 bp upstream of the transcription starting site), TSS200 (200 bp upstream of the transcription starting site), 5’UTR, and 1st exon. We observed significant differences in the distribution percentages of the 439 DMPs on the TSS1500, the TSS200, the 1st Exon, the gene body, and the 3’ UTR as compared with the distribution percentages of the 832,347 CpG sites on each gene region, respectively. While the distribution percentages of the 439 DMPs and the 832,347 CpG sites on the 5’UTR and the intergenic region were not significantly different (**Supplementary Table 7**). Concerning CpG island regions in relation to the 439 DMPs (**Figure 4C**), 350 (79.73%) DMPs were located in the Open Sea (DNA outside the CpG island regions), 15 were located in the CpG island, 43 were located on the CpG shores (0–2 kb upstream and downstream of the CpG island, also called North and South Shore), and 31 were located on the Shelves (2–4 kb upstream and downstream of the CpG island, also called North and South Shelf). Significant differences were observed between the distribution percentages of the 439 DMPs and the 832,347 CpG sites on the island, S shore, and the open sea (**Supplementary Table 8**).

**Table 2:** Comparisons of the distribution percentages of the 439 DMPs and the 832,347 CpG sites on each chromosome.

Chromosome	Distribution percentage of the 439 DMPs (%)	Distribution percentage of the 832,347 CpG sites (%)	*χ* ^2^	*p*-value^#^
chr1	9.57	9.49	0.003	0.956
chr2	8.43	7.51	0.532	0.466
chr3	7.06	5.69	1.528	0.216
chr4	3.19	4.26	1.232	0.267
chr5	5.01	5.19	0.029	0.865
chr6	11.62	6.20	22.147	<0.0001
chr7	5.24	5.40	0.022	0.881
chr8	4.78	4.41	0.147	0.702
chr9	4.33	3.01	2.591	0.107
chr10	6.38	4.82	2.311	0.128
chr11	4.33	5.62	1.378	0.240
chr12	5.47	5.15	0.088	0.766
chr13	0.91	2.44	4.305	0.038
chr14	2.73	3.43	0.646	0.422
chr15	4.10	3.33	0.813	0.367
chr16	1.82	4.39	6.873	0.009
chr17	3.87	5.15	1.462	0.227
chr18	1.82	1.74	0.017	0.897
chr19	1.82	4.49	7.277	0.007
chr20	2.96	2.70	0.117	0.733
chr21	2.51	1.20	6.311	0.012
chr22	0.91	2.13	3.124	0.077
chrX	1.14	2.24	2.418	0.120

DMP, differentially methylated position.

^#^Pearson’s chi-squared test. The significance threshold for each p value is 0.05/23 = 0.002 (for the 23 chromosome groups).

According to the Illumina annotated CpGs and transcript pairs, all CpGs in the Open Sea were excluded from the 439 DMPs. Finally, 313 DMPs including 273 hypomethylated DMPs and 40 hypermethylated DMPs were annotated to genes. As some CpG sites were annotated to more than one gene, 304 DMGs were identified after the duplicate genes were removed. In the 18 DMGs that mapped from more than one CpG site, 15 DMGs were hypomethylated, 2 DMGs were hypermethylated, and only 1 DMG showed mixed methylation (**Supplementary Table 9**). Of the 18 DMGs, when Δ*β* ≥ 0.1, HLA-DPB2, HLA-DRB1, PPP2R5C, and LTF were identified.

**Figure 5:**
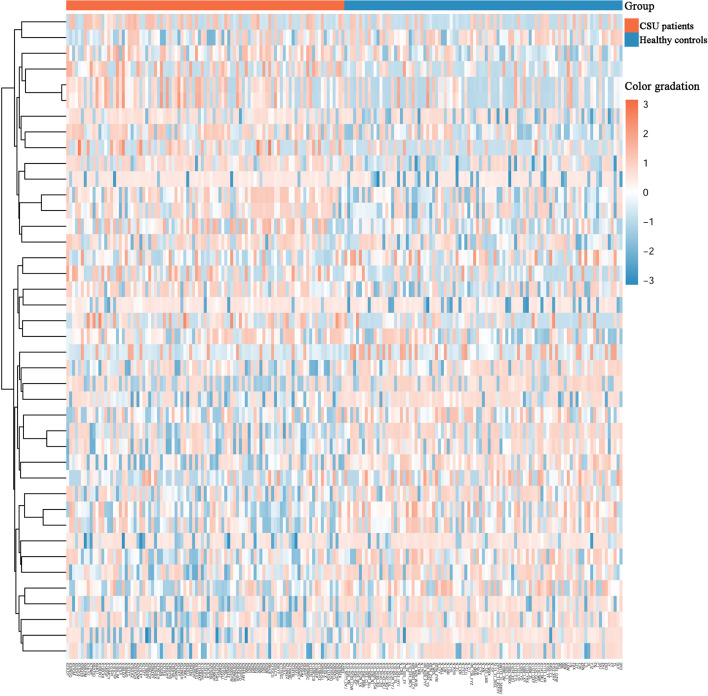
Heatmap of the 41 differentially methylated positions (DMPs) between the CSU patients and the healthy controls (*p* < 0.01 and |Δ*β*| ≥ 0.1). Two-dimensional hierarchical clustering of the 41 DMPs between the 95 CSU patients and the 95 healthy controls. Each row represents an individual DMP and each column represents a participant. There are 41 rows (41 DMPs) and 190 columns (95 CSU patients *vs*. 95 healthy controls) in this figure. On the top, the CSU patients and the healthy controls are represented by red bars and blue bars, respectively. Color gradation from red to blue means from higher methylation levels to lower methylation level. CSU, chronic spontaneous urticaria.

Across the whole genome, the CpG site cg07052231 located on the *PEX5* gene had the highest association with CSU (*p* = 5.16E−13, Δ*β* = −0.08). Compared with the 95 healthy controls, 41 (22 hypomethylated, 19 hypermethylated) DMPs annotated to 28 DMGs (*p* < 0.01 and |Δ*β*| ≥ 0.1) were identified in the 95 CSU patients (**Table 3**). The heatmap of the 41 DMPs between the 95 CSU patients and the 95 healthy controls (*p* < 0.01 and |Δ*β*| ≥ 0.1) is shown in **Figure 5**. The 41 DMP-level differential methylation analysis results (DMP specific violin plots) are shown in **Supplementary Figure 2**.

**Table 3:** The 41 differentially methylated positions (*p* < 0.01 and |Δ*β*| ≥ 0.1) between the CSU patients and the healthy controls.

Probe ID	Δ*β*	*p*-value	DMG	Related disease^#^
cg15184869	−0.185	4.20E−04	PEBP4	IgA nephropathy, neoplasms (breast, lung, ovarian), carcinoma (non-small cell lung, squamous cell), Alzheimer’s disease
cg16241932	−0.138	2.68E−03	ZDHHC14	Diabetes mellitus-related atherosclerosis, neoplasms (prostate, stomach), depressive disorders, lymphoproliferative disorders
cg09456260	−0.134	3.31E−03		
cg09510698	−0.132	5.20E−03	HLA-DPB2	AIDs (ATDs, systemic sclerosis, SLE, Wegener’s granulomatosis, IgA nephropathy), neoplasms (uterine cervix)
cg04083966	−0.130	3.12E−03	MYBPH	Hypertrophic cardiomyopathy, lung squamous cell carcinoma, amyotrophic lateral sclerosis, neurodegenerative diseases
cg06837403	−0.130	2.46E−03		
cg04819054	−0.128	2.75E−03		
cg02018040	−0.126	2.18E−06		
cg13323954	−0.125	4.59E−03		
cg13117487	−0.124	4.65E−03		
cg11401796	−0.118	1.92E−04	C21orf70	Down syndrome
cg01591343	−0.118	8.46E−03	WDR27	Type I diabetes
cg15820961	−0.117	1.08E−04	HLA-DRB1	AID (TID, multiple sclerosis, SLE, RA), HIV/Hepatitis B infections, neoplasms, shared paranoid disorder, sarcoidosis
cg04843821	−0.116	6.23E−03	C10orf68	Neoplasms (colorectal, colon, lung, endometrium), melanoma
cg17600943	−0.116	7.33E−03	CPO	Hereditary coproporphyria, drug-induced liver injury
cg24849373	−0.114	8.94E−03	TSNARE1	Schizophrenia, bipolar disorder
cg23052585	−0.112	5.70E−03		
cg15019001	−0.111	2.82E−04	HLA-DPB2	AIDs (ATDs, systemic sclerosis, SLE, Wegener’s granulomatosis, IgA nephropathy), neoplasms (uterine cervix)
cg04026937	−0.110	9.60E−03	HLA-DRB1	AID (TID, multiple sclerosis, SLE, RA), HIV/hepatitis B infections, neoplasms, shared paranoid disorder, sarcoidosis
cg06013788	−0.101	9.20E−03		
cg05161773	−0.101	7.45E−03	SEPT9	Neoplasms (colorectal, colon, breast, ovarian, prostate), leukemia
cg05865327	−0.101	5.06E−03	PPP2R5C	Neoplasms (lung, colon, breast, renal-cell), melanoma, adenocarcinoma, HIV infection
cg00124902	0.100	6.19E−04	C1orf94	
cg11699126	0.102	3.25E−03	TCF3	Neoplasms (colorectal, colon, breast, ovarian, pancreatic, prostate), precursor cell lymphoblastic leukemia-lymphoma
cg04118610	0.103	2.11E−03	LPHN3	
cg10070328	0.103	6.35E−03		
cg13342259	0.103	4.68E−03		
cg09957458	0.106	2.51E−03		
cg00693253	0.109	4.72E−03	LRP1	Alzheimer’s disease, cerebral amyloid angiopathy, atherosclerosis, neoplasms
cg17113546	0.120	4.92E−04		
cg07437923	0.122	9.37E−03	DNAJA3	Neoplasms (breast, colon, colorectal, uterine cervix), carcinoma (basal cell, non-small cell lung, renal cell, squamous cell)
cg01991743	0.128	3.36E−03	HLA-DPB1	AID (ATD, TID, multiple sclerosis, SLE, RA), HIV/hepatitis B infections, cervical squamous cell carcinoma
cg04331561	0.129	6.85E−03	TLL1	Atrial septal defect, coronary artery disease in TID, hepatocellular carcinoma in hepatitis C virus, multiple sclerosis
cg26889118	0.141	2.19E−03		
cg13177959	0.145	2.33E−03	GCN1	Breast neoplasms, endometriosis
cg17155524	0.152	3.85E−03	ZFYVE28	Schizophrenia, mental disorders, neurodegenerative diseases
cg01427108	0.157	7.37E−05	LTF	AIDs (primary Sjogren’s syndrome, TID, IBDs), infections, metabolic diseases, neoplasms, carcinoma
cg23209941	0.162	5.62E−04	DISC1	Schizophrenia, autistic/bipolar/depressive/mental disorder, Alzheimer diseases, developmental disabilities
cg12179578	0.179	8.08E−05	SLC4A8	Kidney diseases (cystic), tracheomalacia
cg21787089	0.191	2.06E−05	LTF	AIDs (primary Sjogren’s syndrome, TID, IBDs), infections, metabolic diseases, neoplasms, carcinoma
cg14782559	0.198	2.82E−05	COL11A2	AIDs (primary Sjogren’s syndrome, RA, Wegener’s granulomatosis), osteoarthritis, vasculitis

CSU, chronic spontaneous urticaria; DMG, differentially methylated gene; AID, autoimmune disease; ATD, autoimmune thyroid disease; SLE, systemic lupus erythematosus; RA, rheumatoid arthritis; TID, type I diabetes; IBD, inflammatory bowel disease.

^#^Annotation of related diseases was conducted in the Gene-Cloud of Biotechnology Information database.

We divided the 95 CSU patients into eight pairs of subgroups according to eight aspects: elevated/normal tIgE, positive/negative anti-TPO IgG, positive/negative anti-TG IgG, with/without angioedema, refractory/non-refractory, UASday > 4/UASday ≤ 4, disease duration > 6 months/disease duration ≤ 6 months, and recurrent/non-recurrent. Except for the refractory/non-refractory subgroup and the disease duration > 6 months/disease duration ≤ 6 months subgroup, phenotype-specific DMPs were generated in the 439 DMPs in the other six subgroups (**Table 4**).

**Table 4:** Number of differentially methylated positions (DMPs) (*p* < 0.01 and |Δ*β*| ≥ 0.06) in the six pairs of subgroups of CSU patients.

Subgroup (No.)	DMP	Δ*β*	*p* value	Chr.	DMG	Related diseases^#^
**High tIgE** (64) ***vs*. normal tIgE** (31)	cg05001007	−0.137	1.56E−03	10		
	cg08506353	−0.120	2.35E−03	6	HLA-DPB2	AIDs (ATDs, systemic sclerosis, SLE, Wegener’s granulomatosis, IgA nephropathy), neoplasms (uterine cervix)
	cg14782559	0.177	5.23E−03	6	COL11A2	AIDs (primary Sjogren’s syndrome, RA, Wegener’s granulomatosis), osteoarthritis, vasculitis
	cg15019001	−0.122	7.05E−03	6	HLA-DPB2	AIDs (ATDs, systemic sclerosis, SLE, Wegener’s granulomatosis, IgA nephropathy), neoplasms (uterine cervix)
	cg07440017	0.091	7.32E−03	3		
**Anti-TPO +** (18) ***vs*. anti-TPO −** (77)	cg24443559	−0.134	1.90E−03	7	WDR60	Short rib-polydactyly syndrome, osteochondrodysplasias
**Anti-TG +** (24) ***vs*. anti-TG −** (71)	cg12873598	−0.071	3.68E−03	X		
	cg02448295	0.153	1.65E−03	6	BRD2	Melanoma, breast neoplasms, osteosarcoma, leukemia, epilepsy
	cg11720573	−0.132	3.67E−03	15	ABHD2	Chronic obstructive pulmonary disease, selective IgA deficiency, cancers, coronary artery disease
**AE +** (35) ***vs*. AE −** (60)	cg26864304	0.127	7.16E−03	6		
	cg11370814	−0.072	9.84E−03	2		
**UAS ≤ 4** (32) ***vs*. UAS > 4** (63)	cg00917413	−0.083	8.53E−03	7	CAV2	Neoplasms (breast, prostate, thyroid), carcinoma (lung, renal cell), glaucoma, type II diabetes, Alzheimer’s diseases
**Recurrent** (30) ***vs*. non-recurrent** (65)^	cg01992742	−0.114	6.13E−03	20	NCOA3	Neoplasms (breast, prostate, urinary bladder, ovarian, colorectum, lung, brain), carcinoma (hepatocellular, non-small-cell lung, adenocarcinoma)

CSU, chronic spontaneous urticaria; TPO, thyroid peroxidase; TG, thyroglobulin; tIgE, total IgE (high tIgE, tIgE > 100 IU/ml, normal tIgE, tIgE ≤ 100 IU/ml); sIgE, specific IgE; AE, angioedema; DMP, differentially methylated position; Chr., chromosome; DMG, differentially methylated gene; AID, autoimmune disease; ATD, autoimmune thyroid disease; SLE, systemic lupus erythematosus; RA, rheumatoid arthritis.

^#^Annotation of related diseases was conducted in the Gene-Cloud of Biotechnology Information database.

^The average recurrence interval of the 30 recurrent CSU patients was 10.8 (range: 2–30) years.

### Gene Ontology Functional Analysis

Biological process analysis of the 304 DMGs showed that these genes were mainly enriched in regulation of cell signaling, cellular and intracellular compounds’ catabolic and metabolic processes, and cellular response to and regulation of endogenous and exogenous stimulus. Cellular component analysis of the 304 DMGs showed that these genes were significantly enriched in the intracellular part including cytoplasm and organelles. Molecular function analysis of the 304 DMGs showed that these genes were significantly enriched in catalytic activity, protein binding, pyrophosphatase, and hydrolase activity. Gene ontology enrichment analyses in above three aspects were displayed with the top 10 significant *p* values, respectively (**Supplementary Table 10**).

### KEGG Pathway Analysis

The top 30 prominently enriched KEGG pathways of the 304 DMGs are shown in **Table 5**. The DMGs were enriched in the pathways related to autoimmune diseases including IBD, ATD, and TID, as well as important immune-related pathways including antigen processing and presentation, Th17 cell differentiation, Th1 and Th2 cell differentiation, PI3K-Akt signaling pathway, TNF signaling pathway, and AMPK signaling pathway, which play important roles in autoimmune diseases. Among these pathways, the most significant one was the sphingolipid signaling pathway (*p* = 9.08E−05). The pathway with the most number of DMGs was the PI3K-Akt signaling pathway (13 DMGs, *p* = 3.29E−03).

**Table 5:** The top 30 prominently enriched KEGG pathways for the 304 DMGs.

KEGG ID	KEGG pathway	*p*-value	No. of DMG	DMG
hsa04071	Sphingolipid signaling pathway	9.08E−05	9	GNA12/NFKB1/PPP2R5C/MAP3K5/SPTLC2/ROCK2/AKT3/MAPK14/PTEN
hsa05215	Prostate cancer	1.23E−04	8	NFKB1/CREB1/TCF7L2/AKT3/FGFR2/PDGFD/PTEN/TCF7L1
hsa04940	Type I diabetes mellitus	5.02E−04	5	PTPRN2/HLA-DRB1/HLA-DQB1/HLA-C/HLA-DPB1
hsa05166	Human T-cell leukemia virus 1 infection	5.76E−04	11	NFKB1/ANAPC7/CREB1/SMAD4/AKT3/HLA-DRB1/PTEN/HLA-DQB1/HLA-C/TCF3/HLA-DPB1
hsa04659	Th17 cell differentiation	1.33E−03	7	NFKB1/RUNX1/SMAD4/MAPK14/HLA-DRB1/HLA-DQB1/HLA-DPB1
hsa05145	Toxoplasmosis	1.74E−03	7	NFKB1/AKT3/MAPK14/HLA-DRB1/HLA-DQB1/HLA-DPB1/GNAO1
hsa05416	Viral myocarditis	2.31E−03	5	ABL1/HLA-DRB1/HLA-DQB1/HLA-C/HLA-DPB1
hsa04211	Longevity regulating pathway	2.54E−03	6	NFKB1/SESN1/CAMKK2/CREB1/AKT3/RPTOR
hsa05330	Allograft rejection	2.73E−03	4	HLA-DRB1/HLA-DQB1/HLA-C/HLA-DPB1
hsa05321	Inflammatory bowel disease	3.28E−03	5	NFKB1/IL18R1/HLA-DRB1/HLA-DQB1/HLA-DPB1
hsa04151	PI3K-Akt signaling pathway	3.29E−03	13	NFKB1/PPP2R5C/PHLPP1/CREB1/AKT3/MYB/IFNAR2/FGFR2/ITGA1/PDGFD/RPTOR/PTEN/TCL1A
hsa05221	Acute myeloid leukemia	3.51E−03	5	NFKB1/RUNX1/TCF7L2/AKT3/TCF7L1
hsa05332	Graft-*versus*-host disease	3.61E−03	4	HLA-DRB1/HLA-DQB1/HLA-C/HLA-DPB1
hsa05169	Epstein–Barr virus infection	3.99E−03	9	NFKB1/USP7/AKT3/IFNAR2/MAPK14/HLA-DRB1/HLA-DQB1/HLA-C/HLA-DPB1
hsa05164	Influenza A	4.88E−03	8	NFKB1/RAE1/AKT3/IFNAR2/HLA-DRB1/TMPRSS4/HLA-DQB1/HLA-DPB1
hsa05140	Leishmaniasis	6.41E−03	5	NFKB1/MAPK14/HLA-DRB1/HLA-DQB1/HLA-DPB1
hsa05220	Chronic myeloid leukemia	6.41E−03	5	NFKB1/ABL1/RUNX1/SMAD4/AKT3
hsa05152	Tuberculosis	6.63E−03	8	NFKB1/CREB1/AKT3/CLEC7A/MAPK14/HLA-DRB1/HLA-DQB1/HLA-DPB1
hsa04612	Antigen processing and presentation	6.78E−03	5	CREB1/HLA-DRB1/HLA-DQB1/HLA-C/HLA-DPB1
hsa04668	TNF signaling pathway	7.82E−03	6	NFKB1/MAP3K5/IL18R1/CREB1/AKT3/MAPK14
hsa05320	Autoimmune thyroid disease	9.05E−03	4	HLA-DRB1/HLA-DQB1/HLA-C/HLA-DPB1
hsa04152	AMPK signaling pathway	1.08E−02	6	PPP2R5C/CAMKK2/CREB1/ACACA/AKT3/RPTOR
hsa05310	Asthma	1.20E−02	3	HLA-DRB1/HLA-DQB1/HLA-DPB1
hsa05213	Endometrial cancer	1.24E−02	4	TCF7L2/AKT3/PTEN/TCF7L1
hsa04611	Platelet activation	1.26E−02	6	ROCK2/P2RY12/AKT3/PLA2G4A/MAPK14/P2RX1
hsa05130	Pathogenic *Escherichia coli* infection	1.29E−02	8	GNA12/NFKB1/ABL1/MYH10/ROCK2/BAIAP2/MAPK14/MYO10
hsa04658	Th1 and Th2 cell differentiation	1.40E−02	5	NFKB1/MAPK14/HLA-DRB1/HLA-DQB1/HLA-DPB1
hsa04810	Regulation of actin cytoskeleton	1.77E−02	8	GNA12/RDX/MYH10/ROCK2/BAIAP2/FGFR2/ITGA1/PDGFD
hsa05216	Thyroid cancer	1.94E−02	3	TCF7L2/CCDC6/TCF7L1
hsa05131	Shigellosis	2.01E−02	4	NFKB1/ABL1/ROCK2/MAPK14

KEGG, Kyoto Encyclopedia of Genes and Genomes; DMG, differentially methylated gene; ID, identity.

## Discussion

Genome-wide DNA methylation is one of the best-studied mechanisms in the field of epigenetics, which is closely related to the development of autoimmune diseases (4, 5). To the best of our knowledge, this study is the first comprehensive analysis to reveal a distinct genome-wide DNA methylation profile in adult patients with CSU from adult healthy subjects, suggesting the involvement of DNA methylation in the development of CSU.

With regard to the 439 DMPs, CSU patients exhibited significantly lower global methylation levels than those of the 95 healthy controls and 86.6% of the 439 DMPs were hypomethylated. There is increasing evidence that global and focal hypomethylation is an important feature of autoimmune diseases including multiple sclerosis, SLE, and RA (18, 19). The 439 DMPs in the CSU patients distributed widely on autosomal chromosomes. The numbers of these DMPs on different chromosomes varied greatly. Chromosome 6 embodied the largest number and the highest distribution intensity of DMPs whose annotated genes were considerably involved in autoimmunity (20). It has been reported that HLA-DRB1 and HLA-DQB1 are susceptible to CSU (21, 22). Overall, the analysis of the distribution of DMPs across the location distributions revealed that the DMPs were not randomly distributed.

The 41 DMPs (*p* < 0.01 and |Δ*β*| ≥ 0.1) between the CSU patients and the healthy controls are annotated to 28 DMGs and most of the DMGs are related to autoimmune diseases. Interestingly, when |Δ*β*| ≥ 0.1 (simultaneously *p* < 0.01), 4 of the 28 DMGs—HLA-DPB2, HLA-DRB1, PPP2R5C, and LTF, which all mapped from more than one CpG site—are all associated with autoimmunity. Moreover, HLA-DRB1 has been reported to be related to the DNA methylation of multiple autoimmune diseases including Crohn’s disease, multiple sclerosis, SLE, and TID (23–26). A recent study has revealed that the only one DMG with mixed methylation, fibrinogen-like protein 2 (FGFR2), is a mast cell mediator with potential relevance in CSU (27). Desai et al. have demonstrated that IL-6 promotes mast cell maturation and reactivity with downregulation of the suppressor of cytokine signaling 3 autoinhibitory pathway by promotor methylation (28). Their findings explain that CSU might be related to DNA methylation.

Although whole blood contains various mixed cell components, DNA methylation profiles in whole blood are extremely relevant to circulatory biomarkers for autoimmune disorders (29). In this study, leukocyte composition analysis showed the proportions of CD8^+^ T lymphocytes decreased in the CSU patients. Our results are consistent with those of a previous study (30, 31). In our study, the percentage of granulocytes was significantly higher in the blood samples of the CSU patients than that in the blood samples of the healthy controls. With regard to specific type of granulocyte, there is no doubt that basophils and eosinophils play important roles in the pathology of CSU. Basophil histamine release assay (BHRA) and basophil activation test (BAT) aid identification of autoimmune CSU (32). It has been reported that positive BHRA results and low levels of total IgE are predictors of a good response to cyclosporine for CSU (33). However, one of the main pharmacodynamic mechanisms responsible for CSU patients’ clinical response to omalizumab is to rapidly correct the basopenia in CSU patients and downregulate the expression of FcϵRI on basophils (34). On the other side, possible mechanisms of the peripheral blood eosinopenia observed in active CSU patients may include the depletion of blood eosinophils by recruitment into the skin (35). Recent clinical studies have shown that eosinopenia in patients with CSU is associated with type IIb autoimmunity, high disease activity, and poor response to treatment (36).

Compared with corresponding negative phenotype subgroups, CSU patients with elevated tIgE, positive anti-TPO IgG, positive anti-TG IgG, angioedema, UASday > 4, or recurrent CSU possessed phenotype-specific DMPs. Our findings indicate that these phenotype-specific DMPs or DMGs will become potential biomarkers of the corresponding CSU subtypes. According to the study of Altrichter et al., low tIgE may suggest type IIb autoimmune CSU, poor response to treatment with omalizumab, and a better chance to benefit from cyclosporine treatment (37). The study of Kolkhir et al. showed that 28% of CSU patients had at least one autoimmune disease and the most prevalent comorbid autoimmune disease was Hashimoto’s thyroiditis (21%) (38). In our study, CSU patients with comorbid autoimmune diseases were excluded. In our 95 CSU patients, 64 had tIgE > 100 IU/ml and 15 had tIgE < 40 IU/ml, among the 15 low-tIgE patients, only one was positive for anti-TPO IgG AAbs (32). Because autologous serum skin test (ASST), BHRA, BAT, and tests for FcεRI autoantibodies were not performed in this study, the exact numbers of patients with type IIb autoimmune CSU could not be obtained. However, it is obvious that most of the CSU patients in this study were type I autoimmune CSU, not type IIb autoimmune CSU, even though predominant enrichment of the CSU-associated DMGs in immunological pathways was shown in this study. So, our results provide supportive evidence for the immunopathogenesis of CSU mainly based on type I autoimmune CSU patients. Unfortunately, anti-IL24-IgE and anti-dsDNA-IgE were not detected in this study because the related reagents were not commercially available in China.

In this study, gene ontology functional annotation and KEGG pathway analysis showed that the 304 DMGs in the CSU patients involved mainly in the pathways related to immunological functions and autoimmune disorders. Our findings implicate similar pathogenic mechanisms between CSU and other autoimmune disorders (39). We found that sphingolipid signaling pathway was the most significantly clustered pathway associated with CSU. Sphingolipids have emerged as critical structural and signaling molecules that regulate cell growth, survival, signal transduction, immune cell trafficking, and inflammation (40). Multiple sphingolipids produced by antigen-stimulated mast cells exert their complex functions in regulating metabolism through a variety of signaling mechanisms and specific cell surface receptors and in turn fine-tune the maturity and phenotype of mast cells, thereby regulating their reactivity (40, 41). A recent study has indicated that sera of CSU patients induce production of vascular endothelial growth factor in mast cells through the PI3K/Akt/p38MAPK/HIF-1α axis in an IgE-dependent way (42). Activation of the TNF-α/TNF-α receptor signaling pathway has also been reported to be associated with CSU, marked by increased circulating concentrations of TNF-α, sTNF-R1, and sTNF-R2 (43). In this study, significantly enriched pathways of CSU included Th17 cell differentiation as well as Th1 and Th2 cell differentiation pathways. Our findings were consistent with the results of previous studies (44–46). Th2 cells, Th17 cells (44), and Th2 cytokine IL-31 are highly expressed in skin lesions of CSU (47). Th1-/Th2- and Th17-related cytokines are significantly elevated in plasma of CSU patients and correlate with disease activity of CSU (45). We compared our result of the 439 DMPs and the 304 DMGs with previous studies of DNA methylation in whole blood of other allergies or IgE-driven diseases. CpG cg24788483 that annotated to transcription factor 7-like 2 (TCF7L2) was reported to be related to complete remission of asthma (48). The TCF7L2 gene product is a transcription factor that plays a role in the Wnt signaling pathway (49). In addition, we compared our result of the 439 DMPs and the 304 DMGs with previous studies of DNA methylation in whole blood of other autoimmune diseases. Anaparti et al. demonstrated a negative association between differentially methylated CpG in the C6ORF10 gene and RA risk and confirmed differential abundance of C6ORF10 mRNA in patients with RA by qPCR analysis (50). Differentially methylated regions in HLA-DRB1 were observed in CD4^+^ and CD8^+^ T cells purified from peripheral blood of 94 women with multiple sclerosis and 94 healthy women, and differential gene expression for HLA-DRB1 gene was detected in whole blood (51).

Our study has several limitations. First, DNA methylation profile of each cellular component especially basophils and eosinophils in whole blood was not performed in our study. Second, lack of validation of DMPs and gene expression (mRNA) data is another limitation. Moreover, methylation alterations vary greatly across ethnicity, so our findings in Chinese populations may not be universally applicable. So far, DNA methylation studies in other phenotypes of urticaria such as chronic inducible urticaria or CSU patients from other ethnicities and populations have not been carried out. Further studies in different populations are needed to discern the heterogeneity among methylation markers that correlate with CSU. Finally, ASST, BHRA, BAT, anti-FcεRI/IgE autoantibodies, anti-IL24 IgE, and anti-dsDNA IgE were not detected in this study.

## Conclusions

In conclusion, this study provides a preliminary genome-wide DNA methylation profile for Chinese Han ethnicity adult patients with CSU. Our findings provide supportive evidence of immunological pathomechanisms of CSU. Further studies on histone modification and DNA acetylation are needed for exploring the epigenetic pathomechanisms behind CSU.

## Data Availability Statement

The original contributions presented in the study are included in the article/**Supplementary Material**. Further inquiries can be directed to the corresponding author.

## Ethics Statement

The studies involving human participants were reviewed and approved by the Ethics Committee of the First Hospital of China Medical University. The patients/participants provided their written informed consent to participate in this study.

## Author Contributions

TX conceived the study design and funding acquisition. YQ, LZ, and TX recruited participants and prepared samples. YQ, XY, and BT conducted lab experiments. YQ contributed to writing—original draft. TX contributed to writing—review and editing. All authors contributed to the article and approved the submitted version.

## Funding

This study was supported by the Basic Research Project from the Department of Education, Liaoning Province, China (LZ2015078) and the National Key Clinical Specialist Subject Construction Project on Urticaria from National Health Commission [(2012)649].

## Conflict of Interest

XY and BT were employed by Sinotech Genomics Co., Ltd.

The remaining authors declare that the research was conducted in the absence of any commercial or financial relationships that could be construed as a potential conflict of interest.

## Publisher’s Note

All claims expressed in this article are solely those of the authors and do not necessarily represent those of their affiliated organizations, or those of the publisher, the editors and the reviewers. Any product that may be evaluated in this article, or claim that may be made by its manufacturer, is not guaranteed or endorsed by the publisher.
