# Supplementary material for: Genome-Wide DNA Methylation Profile in Whole Blood of Patients With Chronic Spontaneous Urticaria
Source: Front Immunol. 2021 Sep 3;12:681714. doi: 10.3389/fimmu.2021.681714 (PMC8448194; doi:10.3389/fimmu.2021.681714)
Supplement: Supplementary file 1 [file DataSheet_1.docx]

Supplementary Material

**Supplementary Table 1.** Chronic spontaneous urticaria (CSU)-associated genetic variants………………………………………………………………………………....2

**Supplementary Table 2.** Characteristics of the 95 CSU patients and the 95 healthy controls..………………………………………………………………………………..3

**Supplementary Table 3.** Therapeutic regimens of the 36 refractory patients……...4

**Supplementary Table 4.** Therapeutic regimens of the 59 non-refractory patients...6

**Supplementary Table 5.** Characteristics of the 36 refractory cases and the 59 non-refractory cases…………………………………………………………………...7

**Supplementary Table 6.** The 439 differentially methylated postions (DMPs, *p* < 0.01 and |Δβ| ≥ 0.06) in the CSU patients………………….. in a separate Excel file

**Supplementary Table 7.** Comparisons of the distribution percentages of the 439 DMPs and the 832347 CpG sites on each gene region..……………………………...8

**Supplementary Table 8.** Comparisons of the distribution percentages of the 439 DMPs and the 832347 CpG sites in each CpG island region..……………………….9

**Supplementary Table 9.** The 18 differentially methylated genes (DMGs) that mapped from more than one CpG site..……………………………………………...10

**Supplementary Table 10.** The significantly enriched gene ontology terms for the 304 DMGs (top 10 per aspect)………..………………..………….………………...12

**Supplementary Figure 1.** Singular value decomposition (SVD) analysis plot of the confounders……………………..…………………………………………………….13

**Supplementary Figure 2.** The 41 DMPs-specific violin plots………….…………14

**Supplementary Table 1.** Chronic spontaneous urticaria (CSU)-associated genetic variants.

| **Gene** | **SNP/Genetic variant** | **Country** | **Reference** |
| --- | --- | --- | --- |
| IL17RA | rs4819554, rs879577 | Egypt | Nada H, et al. (2020) (1) |
| IL-1 | rs1304037, rs1800587 | Poland | Brzoza Z, et al. (2020) (2) |
| VDR-FokI | rs2228570 | China | Ma Y, et al. (2020) (3) |
| HRH1 | rs901865 | China | Li J, et al. (2020) (4) |
| CRP | rs3093059 | China | Yan S, et al. (2019) (5) |
| CACNA1C | rs58619945, rs216008 | China | Yan J, et al. (2018) (6) |
| IL-2 | rs2069762, rs2069763 | Iran | Movahedi M, et al. (2017) (7) |
| ORAI1 | rs12320939, rs3741596, rs3741595 | China | Li J, et al. (2017) (8) |
| ICOS; CD28 | rs6726035; rs2140148, rs3116496 | Poland | Brzoza Z, et al. (2017) (9) |
| FCER1A | rs2298805 | China | Guo A, et al. (2015) (10) |
| IL-10; TGF-β | rs1800896, rs1800871, rs1800872; rs1982073, rs1800471 | Iran | Tavakol M, et al. (2014) (11) |
| IL-6; TNF-α | rs1800795, nt565G/A; rs800629, rs361525 | Iran | Tavakol M, et al. (2014) (12) |
| IFN-γ; IL-6 | rs2430561; rs1800795 | India | Alasandagutti ML, et al. (2014) (13) |
| CCR2; CCR5 | rs1799864; rs333 (d32) | Poland | Brzoza Z, et al. (2014) (14) |
| C5AR1 | rs11673309 | China | Yan S, et al. (2014) (15) |
| PTPN22 | rs2476601 | Poland | Brzoza Z, et al. (2012) (16) |
| COX-2; ALOX5AP | 5’UTR T/G, Exon 10 T/C; −336 G/A | Italy | Di Lorenzo G, et al. (2011) (17) |
| HLA-A; HLA-B; HLA-DRB1 | *33; *44; *04 | Poland | Bozek A, et al. (2010) (18) |
| HLA-DRB1; HLA-DQB1 | *04, *15; *06, *0302 | U.K. | O’Donnell et al. (1999) (19) |

SNP, single nucleotide polymorphism.

**Supplementary Table 2.** Characteristics of the 95 CSU patients and the 95 healthy controls.

|  | **CSU patients**  **(n = 95)** | **Healthy controls**  **(n = 95)** | ***p* value** |  |
| --- | --- | --- | --- | --- |
| Age, years, median; (IQR) | 36 (29; 46) | 34 (28; 41) | 0.11^#^ |  |
| Sex (female/male) | 69/26 | 76/19 |  |  |
| Female age, years, median; (IQR) | 40 (30.5; 48) | 37 (29; 43.5) | 0.06^#^ |  |
| Male age, years, median; (IQR) | 32 (27.8; 34) | 30 (22; 34) | 0.15^#^ |  |
| Disease duration, months, median; (IQR) | 2.5 (2; 6) | NA | NA |  |
| Angioedema, n (%) | 35 (36.80) | 0 | NA |  |
| UAS, median; (IQR) | 5 (4; 6) | NA | NA |  |
| Total IgE, IU/ml, median; (IQR) | 177.89 (64.08; 308.80) | 0 (0;0) | NA |  |
| Positive serum specific IgE, n (%) | 29 (30.50) | 0 | NA |  |
| Positive anti-TPO/anti-TG IgG autoantibodies, n (%) | 24 (25.30) | 0 | NA |  |
| Positive anti-TPO IgG autoantibodies, n (%) | 18 (18,9) | 0 | NA |  |
| Positive anti-TG IgG autoantibodies, n (%) | 33 (34.7) | 0 | NA |  |

CSU, chronic spontaneous urticaria; IQR, interquartile ranges; UAS, urticaria activity score; TG, thyroglobulin; TPO, thyroid peroxidase; NA: not available.

^#^Mann-Whitney U-test.

**Supplementary Table 3.** Therapeutic regimens of the 36 refractory patients.

| **Dosage** | **n** | **Therapeutic regimen** |
| --- | --- | --- |
| 4-fold dose | 1 | desloratadine 10 mg twice daily |
| 4-fold equivalent dose | 9 |  |
|  | 3 | ebastine 20 mg once daily, olopatadine 5 mg twice daily |
|  | 3 | desloratadine 5 mg, ebastine 20 mg, fexofenadine 180 mg once daily |
|  | 1 | cetirizine 10 mg, ebastine 20 mg, loratadine 10 mg once daily |
|  | 1 | cetirizine 10 mg, ebastine 10 mg, loratadine 10 mg, mizolastine 10 mg once daily |
|  | 1 | ebastine 20 mg once daily, fexofenadine 120 mg thrice daily |
| 4-fold dose / 4-fold equivalent dose and H_2_-antihistamine | 6 |  |
|  | 1 | desloratadine 10 mg twice daily, ranitidine 350 mg twice daily |
|  | 1 | fexofenadine 240 mg thrice daily, ranitidine 350 mg twice daily |
|  | 1 | olopatadine 10 mg twice daily, ranitidine 350 mg twice daily |
|  | 2 | fexofenadine 120 mg thrice daily, olopatadine 5 mg twice daily, ranitidine 350 mg twice daily |
|  | 1 | desloratadine 5 mg, ebastine 20 mg, fexofenadine 180 mg once daily, ranitidine 350 mg twice daily |
| 4-fold equivalent dose and LRA | 5 |  |
|  | 3 | ebastine 20 mg, fexofenadine 180 mg, loratadine 10 mg once daily, montelukast 10 mg once daily |
|  | 1 | desloratadine 10 mg, ebastine 20 mg once daily, montelukast 10 mg once daily |
|  | 1 | ebastine 20 mg once daily, olopatadine 5 mg twice daily, montelukast 10 mg once daily |
| 4-fold dose / 4-fold equivalent dose, H_2_-antihistamine and LRA | 8 |  |
|  | 1 | desloratadine 10 mg twice daily, ranitidine 350 mg twice daily, montelukast 10 mg once daily |
|  | 1 | desloratadine 10 mg twice daily, famotidine 20 mg twice daily, montelukast 10 mg once daily |
|  | 1 | fexofenadine 240 mg thrice daily, ranitidine 350 mg twice daily, montelukast 10 mg once daily |
|  | 1 | olopatadine 10 mg twice daily, ranitidine 350 mg twice daily, montelukast 10 mg once daily |
|  | 2 | ebastine 20 mg once daily, olopatadine 5 mg twice daily, famotidine 20 mg twice daily, montelukast 10 mg once daily |
|  | 1 | desloratadine 5 mg, ebastine 20 mg, fexofenadine 180 mg once daily, ranitidine 350 mg twice daily, montelukast 10 mg once daily |
|  | 1 | desloratadine 5 mg, ebastine 20 mg, loratadine 10 mg once daily, ranitidine 350 mg twice daily, montelukast 10 mg once daily |
| 4-fold dose / 4-fold equivalent dose, H_2_-antihistamine and Chinese traditional medication | 2 |  |
|  | 1 | olopatadine 10 mg twice daily, ranitidine 350 mg twice daily, TGP 2 capsules thrice daily |
|  | 1 | ebastine 20 mg once daily, olopatadine 5 mg twice daily, famotidine 20 mg twice daily, TGP 2 capsules thrice daily |
| 4-fold dose, LRA and Chinese traditional medication | 1 | fexofenadine 240 mg thrice daily, montelukast 10 mg once daily, TGP 2 capsules thrice daily |
| 4-fold dose, H_2_-antihistamine, LRA and Chinese traditional medication | 4 |  |
|  | 1 | desloratadine 10 mg twice daily, ranitidine 350 mg twice daily, montelukast 10 mg once daily, TGP 2 capsules thrice daily |
|  | 1 | levocetirizine 10 mg twice daily, ranitidine 350 mg twice daily, montelukast 10 mg once daily, TGP 2 capsules thrice daily |
|  | 1 | olopatadine 10 mg twice daily, famotidine 20 mg twice daily, montelukast 10 mg once daily, TGP 2 capsules thrice daily |
|  | 1 | olopatadine 10 mg twice daily, ranitidine 350 mg twice daily, montelukast 10 mg once daily, TGP 2 capsules thrice daily |

LRA, leukotriene receptor antagonist; TGP, total glucosides of paeony.

**Supplementary Table 4.** Therapeutic regimens of the 59 non-refractory patients.

| **Dosage** | **n** | **Therapeutic regimen** |
| --- | --- | --- |
| licensed dose | 24 | fexofenadine 180 mg once daily, n=9; cetirizine 10 mg once daily, n=4; ebastine 10 mg once daily, n=4; loratadine 10 mg once daily, n=4; desloratadine 5 mg once daily, n=1; levocetirizine 5 mg once daily,n=1; mizolastine 10 mg once daily, n=1 |
| 2-fold dose | 11 | ebastine 20 mg once daily, n=9; olopatadine 5 mg twice daily, n=2 |
| 2-fold equivalent dose | 9 | desloratadine 5 mg, loratadine 10 mg once daily, n=4; cetirizine 10 mg, fexofenadine 180 mg once daily, n=2; fexofenadine 180 mg, loratadine 10 mg once daily, n=2; cetirizine 10 mg, loratadine 10 mg once daily, n=1 |
| 3-fold equivalent dose | 15 | ebastine 20 mg, fexofenadine 180 mg once daily, n=12; ebastine 20 mg, loratadine 10 mg once daily, n=2; desloratadine 5 mg, fexofenadine 180 mg and loratadine 10 mg once daily, n=1 |

**Supplementary Table 5.** Characteristics of the 36 refractory cases and the 59 non-refractory cases.

|  | **Refractory (n=36)** | **Non-refractory (n=59)** | ***p* value** |
| --- | --- | --- | --- |
| Age, years, median; (IQR) | 34(30; 40.8) | 39 (28; 48) | 0.27^#^ |
| Sex, n (%) |  |  |  |
| Female | 20 (55.6) | 49 (83.1) | 0.01^ |
| Male | 16 (44.4) | 10 (16.9) |  |
| Disease duration, months, median; (IQR) | 2 (1.6; 8.8) | 3 (2; 5.5) | 0.30^#^ |
| Angioedema, n (%) |  |  |  |
| With | 13 (36.1) | 22 (37.3) | 0.91^ |
| Without | 23 (63.9) | 37 (62.7) |  |
| UAS, median; (IQR) | 5 (4; 6) | 5 (4; 6) | 0.40^#^ |
| Total IgE, IU/mL, median; (IQR) | 178.15 (83.35; 322.15) | 177.89 (47.29; 308.80) | 0.85^#^ |
| Anti-TPO IgG autoantibodies, n (%) |  |  |  |
| Positive | 3 (8.3) | 15 (25.4) | 0.04^ |
| Negative | 33 (91.7) | 44 (74.6) |  |
| Anti-TG IgG autoantibodies, n (%) |  |  |  |
| Positive | 8 (22.2) | 16 (27.1) | 0.59^ |
| Negative | 28 (77.8) | 43 (72.9) |  |
| Serum specific IgE, n (%) |  |  |  |
| Positive | 12 (33.3) | 21 (35.6) | 0.82^ |
| Negative | 24 (66.7) | 38 (64.4) |  |

CSU, chronic spontaneous urticaria; IQR, interquartile ranges; UAS, urticaria activity score; TPO, thyroid peroxidase; TG, thyroglobulin.

^#^Mann-Whitney U-test.

^Pearson's chi-squared test.

**Supplementary Table 7.** Comparisons of the distribution percentages of the 439 DMPs and the 832347 CpG sites on each gene region.

| **Gene region** | **distribution percentage of the 439 DMPs (%)** | **distribution percentage of the 832 347 CpG sites (%)** | **χ^2^** | ***p* value^#^** |
| --- | --- | --- | --- | --- |
| TSS1500 | 4.95 | 12.70 | 16.090 | < 0.0001 |
| TSS200 | 0.86 | 7.89 | 32.295 | < 0.0001 |
| 5'UTR | 8.82 | 11.13 | 2.902 | 0.088 |
| 1^st^Exon | 0.65 | 4.66 | 17.269 | < 0.0001 |
| Body | 52.90 | 36.31 | 50.356 | < 0.0001 |
| 3'UTR | 4.73 | 2.46 | 9.397 | 0.002 |
| IGR | 27.10 | 24.84 | 0.818 | 0.366 |

DMP, differentially methylated position.

**^#^**Pearson's chi-squared test. The significance threshold for each *p* value is 0.05/7 = 0.007 (for the 7 gene region groups).

**Supplementary Table 8.** Comparisons of the distribution percentages of the 439 DMPs and the 832347 CpG sites in each CpG island region.

| **Island region** | **distribution percentage of the 439 DMPs (%)** | **distribution percentage of the 832 347 CpG sites (%)** | **χ^2^** | ***p* value^#^** |
| --- | --- | --- | --- | --- |
| North Shelf | 2.51 | 3.71 | 1.771 | 0.183 |
| North Shore | 6.38 | 9.64 | 5.365 | 0.021 |
| Island | 3.42 | 18.18 | 64.288 | < 0.0001 |
| South Shore | 3.42 | 8.23 | 13.478 | < 0.0001 |
| South Shelf | 4.56 | 3.43 | 1.664 | 0.197 |
| Open Sea | 79.73 | 56.81 | 93.935 | < 0.0001 |

DMP, differentially methylated position.

**^#^**Pearson's chi-squared test. The significance threshold for each *p* value is 0.05/6 = 0.008 (for the 6 island region groups).

**Supplementary Table 9.** The 18 differentially methylated genes (DMGs) that mapped from more than one CpG site.

| **Methylation level** | **DMG** | **DMP** | **Δβ** | ***p* value** |
| --- | --- | --- | --- | --- |
| Hypomethylation | APP | cg01154210 | -0.061 | 1.16E-07 |
|  |  | cg19423170 | -0.068 | 7.11E-06 |
|  | ERICH1 | cg07889790 | -0.074 | 5.28E-04 |
|  |  | cg17373649 | -0.097 | 6.21E-03 |
|  | HLA-DPB2 | cg15019001 | -0.111 | 2.82E-04 |
|  |  | cg08506353 | -0.088 | 1.30E-03 |
|  |  | cg09510698 | -0.132 | 5.20E-03 |
|  | HLA-DQB1 | cg21493951 | -0.080 | 9.55E-04 |
|  |  | cg01745539 | -0.063 | 1.96E-03 |
|  |  | cg10180404 | -0.083 | 2.19E-03 |
|  | HLA-DRB1 | cg15820961 | -0.117 | 1.08E-04 |
|  |  | cg04026937 | -0.110 | 9.60E-03 |
|  | HUWE1 | cg04732279 | -0.063 | 8.09E-06 |
|  |  | cg11887420 | -0.066 | 1.74E-05 |
|  | LINC01581 | cg07219453 | -0.073 | 5.52E-08 |
|  |  | cg06386426 | -0.061 | 4.17E-07 |
|  | LOC101926913 | cg17042723 | -0.061 | 1.19E-08 |
|  |  | cg04790775 | -0.065 | 2.98E-08 |
|  | MARK3 | cg03718013 | -0.068 | 3.90E-08 |
|  |  | cg13394762 | -0.064 | 5.60E-08 |
|  | PPP2R5C | cg22954437 | -0.066 | 7.30E-09 |
|  |  | cg05865327 | -0.101 | 5.06E-03 |
|  | RFX3 | cg17787809 | -0.062 | 1.24E-08 |
|  |  | cg08347063 | -0.062 | 2.66E-08 |
|  |  | cg05417534 | -0.061 | 1.16E-06 |
|  | ROCK2 | cg11427511 | -0.063 | 9.76E-08 |
|  |  | cg26300092 | -0.063 | 1.82E-07 |
|  | SESN1 | cg13846195 | -0.063 | 1.16E-08 |
|  |  | cg03511624 | -0.060 | 1.37E-08 |
|  | SPTLC2 | cg06411521 | -0.068 | 4.35E-08 |
|  |  | cg13713667 | -0.063 | 3.58E-07 |
|  | TCF7L2 | cg17150069 | -0.063 | 3.27E-07 |
|  |  | cg02379348 | -0.060 | 4.37E-07 |
| Hypermethylation | HLA-C | cg13872627 | 0.074 | 1.92E-03 |
|  |  | cg11574174 | 0.089 | 8.97E-03 |
|  | LTF | cg21787089 | 0.191 | 2.06E-05 |
|  |  | cg01427108 | 0.157 | 7.37E-05 |
| Mixed methylation | FGFR2 | cg01981452 | -0.063 | 1.69E-06 |
|  |  | cg08899523 | -0.070 | 4.72E-04 |
|  |  | cg16653991 | -0.076 | 1.14E-03 |
|  |  | cg10379346 | 0.072 | 2.10E-03 |

DMP, differentially methylated position.

**Supplementary Table 10.** The significantly enriched gene ontology terms for the 304 DMGs (top 10 per aspect).

| **GO ID** | **GO term** | ***p* value** | **No. of DMG** |
| --- | --- | --- | --- |
| **Biological process** | | | |
| GO:1902531 | regulation of intracellular signal transduction | 3.30E-07 | 50 |
| GO:1901701 | cellular response to oxygen-containing compound | 4.90E-07 | 34 |
| GO:1901700 | response to oxygen-containing compound | 4.60E-06 | 42 |
| GO:0006796 | phosphate-containing compound metabolic process | 6.40E-06 | 90 |
| GO:0044248 | cellular catabolic process | 6.80E-06 | 75 |
| GO:0009056 | catabolic process | 8.10E-06 | 80 |
| GO:0009966 | regulation of signal transduction | 8.60E-06 | 69 |
| GO:0006793 | phosphorus metabolic process | 1.20E-05 | 90 |
| GO:0035556 | intracellular signal transduction | 2.10E-05 | 61 |
| GO:0070887 | cellular response to chemical stimulus | 2.20E-05 | 65 |
| **Molecular function** | | | |
| GO:0003824 | catalytic activity | 1.40E-04 | 133 |
| GO:0005515 | protein binding | 3.30E-04 | 186 |
| GO:0016462 | pyrophosphatase activity | 3.80E-04 | 40 |
| GO:0016817 | hydrolase activity, acting on acid anhydrides | 3.90E-04 | 40 |
| GO:0016818 | hydrolase activity, acting on acid anhydrides, in phosphorus-containing anhydrides | 3.90E-04 | 40 |
| GO:0016787 | hydrolase activity | 5.30E-04 | 77 |
| GO:0030145 | manganese ion binding | 7.60E-04 | 5 |
| GO:0042605 | peptide antigen binding | 8.10E-04 | 4 |
| GO:0017111 | nucleoside-triphosphatase activity | 1.61E-03 | 37 |
| GO:0000287 | magnesium ion binding | 1.92E-03 | 9 |
| **Cellular component** | | | |
| GO:0005737 | cytoplasm | 2.70E-09 | 200 |
| GO:0044424 | intracellular part | 4.00E-08 | 231 |
| GO:0044444 | cytoplasmic part | 7.30E-08 | 172 |
| GO:0005622 | intracellular | 2.90E-07 | 232 |
| GO:0043226 | organelle | 1.90E-06 | 215 |
| GO:0043231 | intracellular membrane-bounded organelle | 2.00E-06 | 196 |
| GO:0044446 | intracellular organelle part | 2.00E-06 | 164 |
| GO:0043229 | intracellular organelle | 2.10E-06 | 208 |
| GO:0043227 | membrane-bounded organelle | 2.80E-06 | 205 |
| GO:0044422 | organelle part | 4.80E-06 | 164 |

GO, gene ontology; ID, identify; DMG, differentially methylated gene.

**Supplementary Figure 1.** Singular value decomposition (SVD) analysis plot of the confounders

**
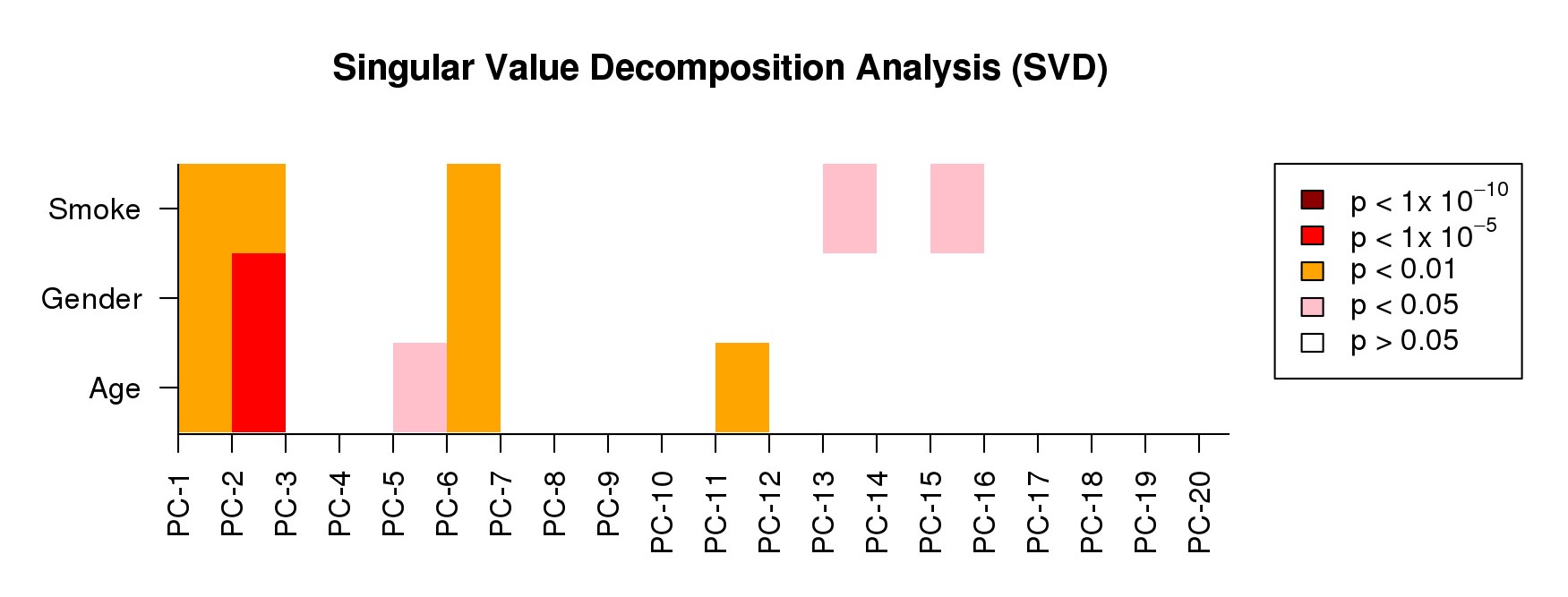
**

The result of SVD analysis is a heatmap of the top principal components correlated to the confounders including age, sex and smoking status. In the heatmap, the darker colours represent a more significant p value, indicating a stronger correlation of the principal component with confounders. From the statistical p values corresponding to the color block in the figure, age, sex and smoking status show significant correlation with DNA methylation of the samples.

**Supplementary Figure 2**. The 41 DMPs-specific violin plots


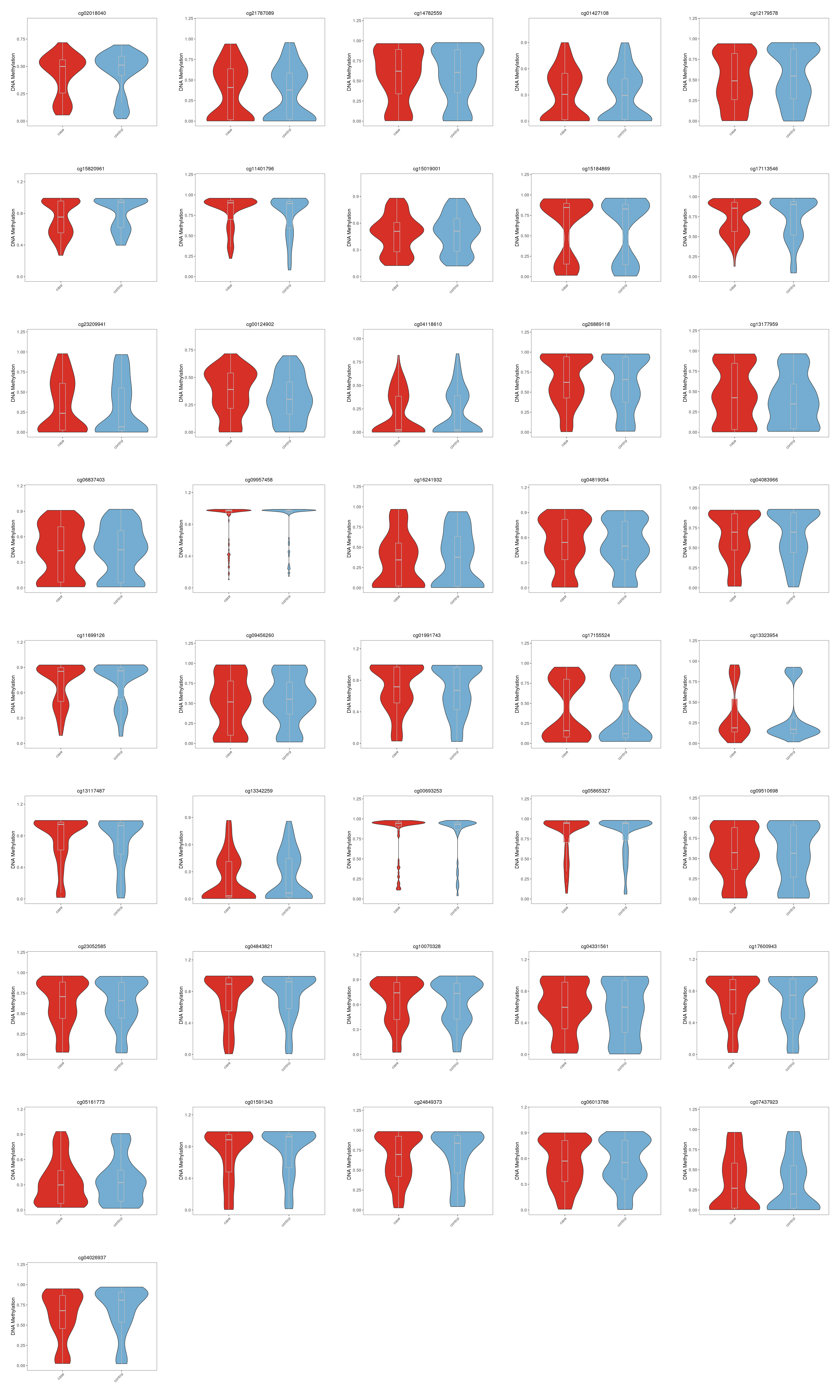


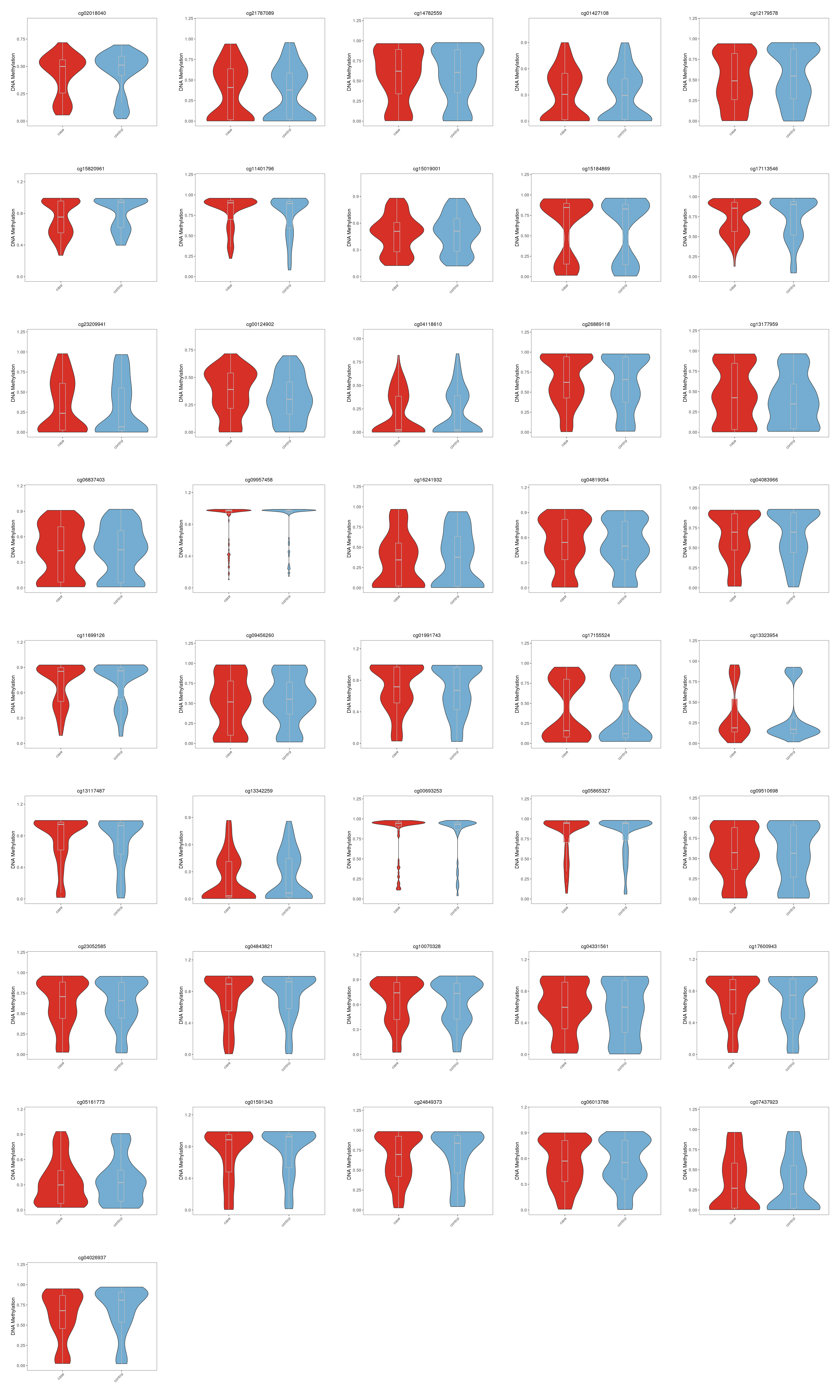


The level differential methylation analysis results of the 41 DMPs (p < 0.01 and |Δβ| ≥ 0.1) between the 95 CSU patients and the 95 healthy controls. Each plot represents the DNA methylation level of a single DMP between the 95 CSU patients (red) and the 95 healthy controls (blue).

**References**

1. Nada H, Hassan R, Ibrahim RAE, Abdelsalam OE, Fathy A, Toraih EA, et al. Interleukin 17 receptor A haplotype analysis in chronic spontaneous urticaria: A preliminary study. *J Cosmet Dermatol*. (2020). doi: 10.1111/jocd.13730

2. Brzoza Z, Rymarczyk B, Grzeszczak W, Trautsolt W, Pluta-Kubicz M, Moczulski D. Interleukin 1 Gene Polymorphisms Presumably Participate in the Pathogenesis of Chronic Spontaneous Autoreactive Urticaria. *J Interferon Cytokine Res*. (2020) 40(10):497-500. doi: 10.1089/jir.2020.0103

3. Ma Y, Xiang Z, Yao X, Li C, Wu J, Feng S, et al. Associations between vitamin D receptor gene polymorphisms and chronic spontaneous urticaria in Chinese Han population. *Postepy Dermatol Alergol*. (2020) 37(2):250-4. doi: 10.5114/ada.2020.94843

4. Li J, Chen W, Peng C, Zhu W, Liu Z, Zhang W, et al. Human H1 receptor (HRH1) gene polymorphism is associated with the severity of side effects after desloratadine treatment in Chinese patients with chronic spontaneous uticaria. *Pharmacogenomics J*. (2020) 20(1):87-93. doi: 10.1038/s41397-019-0094-0

5. Yan S, Chen W, Peng C, Zhu W, Chen M, Zhang J, et al. C-reactive protein (CRP) rs3093059C predicts poor mizolastine response in chronic spontaneous urticaria patients with elevated serum CRP level. *Exp Dermatol*. (2019) 28(3):240-6. doi: 10.1111/exd.13874

6. Yan J, Li Q, Luo Y, Yan S, He Y, Chen X. [Association of CACNA1C gene genetic polymorphism with the susceptibility as well as prognosis for chronic spontaneous urticaria]. *Zhong Nan Da Xue Xue Bao Yi Xue Ban*. (2018) 43(9):929-36. doi: 10.11817/j.issn.1672-7347.2018.09.001

7. Movahedi M, Tavakol M, Rahmani F, Amirzargar AA, Bidoki AZ, Heidari K, et al. Single nucleotide polymorphisms of IL-2, but not IL-12 and IFN-γ, are associated with increased susceptibility to chronic spontaneous urticaria. *Allergol Immunopathol (Madr)*. (2017) 45(4):333-8. doi: 10.1016/j.aller.2016.10.009

8. Li J, Guo A, Chen W, Bin L, He Y, Zhu W, et al. Association of ORAI1 gene polymorphisms with chronic spontaneous urticaria and the efficacy of the nonsedating H1 antihistamine desloratadine. *J Allergy Clin Immunol*. (2017) 139(4):1386-8.e9. doi: 10.1016/j.jaci.2016.10.017

9. Brzoza Z, Grzeszczak W, Trautsolt W, Moczulski D. Inducible T-cell costimulator (ICOS) and CD28 polymorphisms possibly play a role in the pathogenesis of chronic autoreactive urticaria. *Clin Exp Dermatol*. (2017) 42(8):863-7. doi: 10.1111/ced.13212

10. Guo A, Zhu W, Zhang C, Wen S, Chen X, Chen M, et al. Association of FCER1A genetic polymorphisms with risk for chronic spontaneous urticaria and efficacy of nonsedating H1-antihistamines in Chinese patients. *Arch Dermatol Res*. (2015) 307(2):183-90. doi: 10.1007/s00403-014-1525-z

11. Tavakol M, Movahedi M, Amirzargar AA, Aryan Z, Bidoki AZ, Heidari K, et al. Association of interleukin 10 and transforming growth factor β gene polymorphisms with chronic idiopathic urticaria. *Acta Dermatovenerol Croat*. (2014) 22(4):239-45.

12. Tavakol M, Amirzargar AA, Movahedi M, Aryan Z, Bidoki AZ, Gharagozlou M, et al. Interleukin-6 and tumor necrosis factor-alpha gene polymorphisms in chronic idiopathic urticaria. *Allergol Immunopathol (Madr)*. (2014) 42(6):533-8. doi: 10.1016/j.aller.2013.06.004

13. Alasandagutti ML, Ponnana M, Sivangala R, Thada S, Joshi L, Hussain H, et al. Role of IFN-γ and IL-6 cytokines and their association in determining susceptibility to chronic idiopathic urticaria. *Genet Test Mol Biomarkers*. (2014) 18(12):804-9. doi: 10.1089/gtmb.2014.0193

14. Brzoza Z, Grzeszczak W, Rogala B, Trautsolt W, Moczulski D. Possible contribution of chemokine receptor CCR2 and CCR5 polymorphisms in the pathogenesis of chronic spontaneous autoreactive urticaria. *Allergol Immunopathol (Madr)*. (2014) 42(4):302-6. doi: 10.1016/j.aller.2013.02.003

15. Yan S, Chen W, Wen S, Zhu W, Guo A, Chen X, et al. Influence of component 5a receptor 1 (C5AR1) -1330T/G polymorphism on nonsedating H1-antihistamines therapy in Chinese patients with chronic spontaneous urticaria. *J Dermatol Sci*. (2014) 76(3):240-5. doi: 10.1016/j.jdermsci.2014.09.012

16. Brzoza Z, Grzeszczak W, Rogala B, Trautsolt W, Moczulski D. PTPN22 polymorphism presumably plays a role in the genetic background of chronic spontaneous autoreactive urticaria. *Dermatology*. (2012) 224(4):340-5. doi: 10.1159/000339332

17. Di Lorenzo G, Pacor ML, Candore G, Listi F, Ditta V, Leto-Barone MS, et al. Polymorphisms of cyclo-oxygenases and 5-lipo-oxygenase-activating protein are associated with chronic spontaneous urticaria and urinary leukotriene E4. *Eur J Dermatol*. (2011) 21(1):47-52. doi: 10.1684/ejd.2011.1163

18. Bozek A, Krajewska J, Filipowska B, Polanska J, Rachowska R, Grzanka A, et al. HLA status in patients with chronic spontaneous urticaria. *Int Arch Allergy Immunol*. (2010) 153(4):419-23. doi: 10.1159/000316354

19. O'Donnell BF, O'Neill CM, Francis DM, Niimi N, Barr RM, Barlow RJ, et al. Human leucocyte antigen class II associations in chronic idiopathic urticaria. *Br J Dermatol*. (1999) 140(5):853-8. doi: 10.1046/j.1365-2133.1999.02815.x
